# Effect of COVID-19 pandemic on the implementation of a multimorbidity person-centered care model: A qualitative study from health teams’ perspective

**DOI:** 10.1371/journal.pone.0265091

**Published:** 2022-03-22

**Authors:** Paula Zamorano, Alvaro Tellez, Paulina Muñoz, Jaime C. Sapag, Mayra Martinez

**Affiliations:** 1 Centro de Innovación en Salud ANCORA UC, Facultad de Medicina, Pontificia Universidad Católica de Chile, Santiago, Chile; 2 Health Technology Assessment Unit, Center of Clinical Research, Pontificia Universidad Católica de Chile, Santiago, Chile; 3 Department of Family Medicine, Pontificia Universidad Católica de Chile, Santiago, Chile; 4 Department of Public Health, Pontificia Universidad Católica de Chile, Santiago, Chile; 5 Dalla Lana School of Public Health, University of Toronto, Toronto, Canada; Queensland University of Technology, AUSTRALIA

## Abstract

The COVID-19 pandemic has abruptly changed care priority and delivery, delaying others like the multimorbidity approach. The Centro de Innovación en Salud ANCORA UC, the Health National Fund, and the Servicio de Salud Metropolitano Sur Oriente implemented a Multimorbidity Patient-Centered Care Model as a pilot study in the public health network from 2017 to 2020. Its objective was to reorganize the single diagnosis standard care into a new one based on multimorbidity integrated care. It included incorporating new roles, services, and activities according to each patient’s risk stratification. This study aims to describe the perception of the health care teams regarding the impact of the COVID-19 pandemic on four main topics: how the COVID-19 pandemic affected the MCPM implementation, how participants adapted it, lessons learned, and recommendations for sustainability. We conducted a qualitative study with 35 semi-structured interviews between October and December 2020. Data analysis was codified, triangulated, and consolidated using MAXQDA 2020. Results showed that the pandemic paused the total of the implementation practically. Positive effects were the improvement of remote health care services, the activation of self-management, and the cohesion of the teamwork. In contrast, frequent abrupt changes and reorganization forced by pandemic evolution were negative effects. This study revealed the magnitude of the pandemic in the cancelation of health services and identified the urgent need to restart chronic services incorporating patient-centered care in our system.

## Introduction

The covid-19 pandemic has been severe in Latin America, where COVID-19 cases doubled every two days [1]. It has forced the dismantling of the traditional care system and organized one adjusted to the new demands, postponing other essential services, for non-communicable chronic diseases (NCDs) and other health condition [2, 3]. The main reason for this interruption was that clinical staff was reassigned for COVID-19 response (50%) and cancelation of elective care (58%) as well as face-to-face clinical services (50%) [2] In Chile, the evolution of the pandemic has been similar to other countries. More than 1,6 million people (7,6% of the total population) have been infected with COVID-19, and more than 35,000 have died until August 2021. The Metropolitan Region concentrates almost 40% of the cases, being more frequent in men and persons aged 60 and over [4].

In Chile, the public health system offers care for almost 80% of the population, whereas the rest is provided by the private sector [5]. The Ministry of Heath implemented several strategies to face the pandemic, like a broad reorganization of public and private health services, incorporation of traceability, and centralized control of hospitalization beds. As a result, some services were strongly potentiated, like telemedicine, and others faced severe restrictions such as elective surgeries and clinical consultations [6, 7]. Despite the local government has established practical guidelines to reduce the pandemic impact on morbidity, complications, and mortality from NCDs [8], the focus is still on one big problem, COVID-19, which is unsustainable over time for Chile but also for other countries.

**Fig 1:**
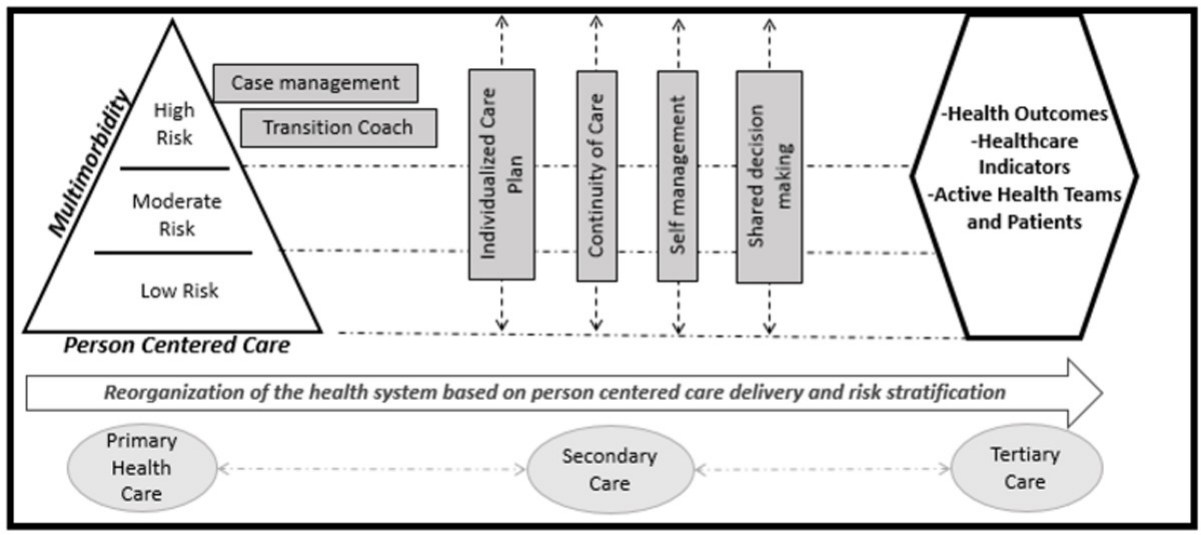
Multimorbidity patient centered care model.

The Centro de Innovación en Salud ANCORA UC (CISAUC), together with the Servicio de Salud Metropolitano Sur Oriente (SSMSO) and the National Health Fund (FONASA), before the pandemic, implemented as a pilot study a Multimorbidity Person-Centered Care (MCPM) model in primary care centers and hospitals (Fig 1). This model aimed to change and reorganize the single diagnosis approach to a new one based on patient-centered care, risk stratification, self-management, case management, individualized care plans, shared responsibility, and continuity of care. These strategies has shown to be today’s best approach for people with multimorbidity, where high-quality care that includes personal needs could potentially be more effective and efficient than the single diagnosis approach [9, 10]. Hence, it is expected to reduce the complications of NCDs, use health services, and improve the quality of life [11].

The implementation of the MCPM began in early 2017 and continued until the end of 2020. Based on the core elements mentioned before, interventions strategies were designed and implemented according to each person’s risk. For example, for high-risk persons, case management was provided with a nurse case manager, a nurse technician, and a physician that provided frequent face-to-face clinical care and distance health counseling, education on self-management of chronic diseases, utilization of the health network, and others. For moderate and low risk, clinical services were provided according to each person’s needs with a strong focus on telemedicine and phone follow-up. Self-management had a strong role in these patients, focusing on continuously improving patient skills.

The pandemic hit the piloting at the stage of consolidation of the interventions, where the new roles, health services, and activities were already rolling out and being established. Unfortunately, and like all other care services, the clinical care for non-communicable diseases was postponed affecting the consolidation process. It has directly interrupted the continuity of care, affecting timely consultations, leaving people with uncovered health needs [6]. This pilot was not an exemption and directly affected the implementation, consolidation, and sustainability work done with the local teams.

Implementing a model like this implied organizational, structural, and cultural changes. The reorganization involved incorporating defined intervention strategies differentiated according to each person’s multimorbidity risk/complexity, which meant the adaption of several core activities such as scheduling consultations, programing future clinical assignments, adaption registration protocols, consultation times, etc. The work done during the implementation process allowed us to systematize this experience and consolidate the minimal conditions for implementation and scale-up [12], which were used as the base for today’s Ministry of Health strategy that is being scaled up [13].

During the third year of piloting, the spread of COVID-19 defied the local health teams and health services on the implementation and consolidation of the model. The objective of this article is to describe health teams’ perception regarding the impact of the COVID-19 pandemic on the implementation of Multimorbidity Person-Centered Care (MCPM) in SSMSO.

## Materials and methods

A qualitative study was performed with data collected between October and December 2020 from 13 individual and 22 group interviews (two to five participants per group). Multidisciplinary health care teams were interviewed from the seven primary care centers (PHC/CESFAM), three hospitals, and local managers where the MCMP was implemented. Intervened PHC and the secondary and tertiary centers offered health services to a population of the southeast of Santiago belonging to the SSMSO. The participating institutions were the following: Centro de Salud Familiar (CESFAM) La Florida, La Florida; CESFAM Villa O´Higgins, La Florida; CESFAM Santiago Nueva Extremadura, La Pintana; CESFAM El Roble, La Pintana; ANCORA UC Madre Teresa Calcuta; ANCORA UC San Alberto Hurtado; ANCORA UC Juan Pablo II. Hospital La Florida, Hospital Dr. Sotero del Rio and Hospital Padre Hurtado.

Participants for the study considered: decision-makers and management teams, health teams at primary care and transition care nurses at hospitals, and implementation teams of CISAUC and SSMSO. The applied inclusion criteria were the following: have participated in implementing the MCPM, performed intervention strategies or management activities. The selection was intentional, considering participants from all the pilot centers in primary care and hospitals. We organized them into groups according to their role throughout implementation.

The data collection tool was the semi-structured interview. An external team from the Public Health Department of the Pontificia Universidad Católica performed the interviews in Spanish and then provided the translations for this study. The researchers designed three main questions and other sub-questions based on the Implementation Evaluation Model of the CISAUC [14]. They looked for the perception of both internal and external context. They addressed the following topics: implementation process, patient satisfaction, and identification of experiences from the piloting that contribute to scalability. During the interview process, the impact of pandemic COVID-19 in implementing the MCPM was identified as an emerging topic, so additional questions were developed to deepen this subject. Those question were the following: (1) Do you think that the pandemic context influenced the implementation/consolidation of MCPM; (2) In what way and how?; (3)What would you suggest to adapt on the current context of the MCPM?

Interviews were coordinated with local’s implementation leaders or directly with individuals, in the case of single interviews. They were conducted remotely, recorded, and executed by two experienced persons in qualitative research techniques. Each interview lasted 60 minutes approximately. After, they were *verbatim* transcribed for analysis. From the 35 interviews, 29 were done with PHC teams, two with nurse transition coaches at hospitals, two with decision-makers at the SSMSO, and two with the CISAUC team.

Data analysis was carried out using the *verbatim* transcriptions of the 35 interviews. The codification process was done simultaneously with the data recollection to facilitate the incorporation of the emerging topics and reach data saturation. A mixed codification was done, elaborating analysis categories that consider both MCMP core elements and emerging information. Finally, the information was categorized according to its properties and dimensions. For this, MAXQDA 2020 was used.

During the analysis process, interpretative triangulation of the data was carried out to increase the reliability and quality of the study process. Two evaluators read the interviews and compared coding to ensure the same interpretation of the evaluative categories. Analysis was conducted until saturation, at which no new themes emerged [15].

The study has been approved by the ethical committees of the Pontificia Universidad Católica de Chile and SSMSO, ID 200717004: “Evaluación de la Implementación y Satisfacción Usuaria en el Modelo de Atención Centrado en la Persona con morbilidad Crónica (MACEP), en el Servicio de Salud Metropolitano Sur Oriente”. The ethics committee waived the need for informed consent given that it didn’t use sensitive identifiable data, nor change in the clinical behavior of the participants, nor need to contact the participants for additional information.

## Results

A total of 69 persons were interviewed (Table 1). The participants were either health professionals or technicians. There were nurses, general practitioners, family physicians, nutritionists, nurse technicians, physiotherapists, and psychologists between these. Regarding gender, 52 (75%) were women and 17 (25%) men.

**Table 1:** Description of the participants.

Participants	Number of persons	Location
Decision-makers and management team	16 (23%)	PHC
Health care teams	42 (61%)	PHC
Transition coach nurses	3 (4%)	Hospitals
Implementation team	8 (12%)	CISAUC and SSMSO

** PHC: Primary Health Centers; CISAUC: Centro de Innovación en Salud ANCORA UC; SSMSO: Servicio de Salud Metropolitano Sur Oriente.

**Fig 2:**
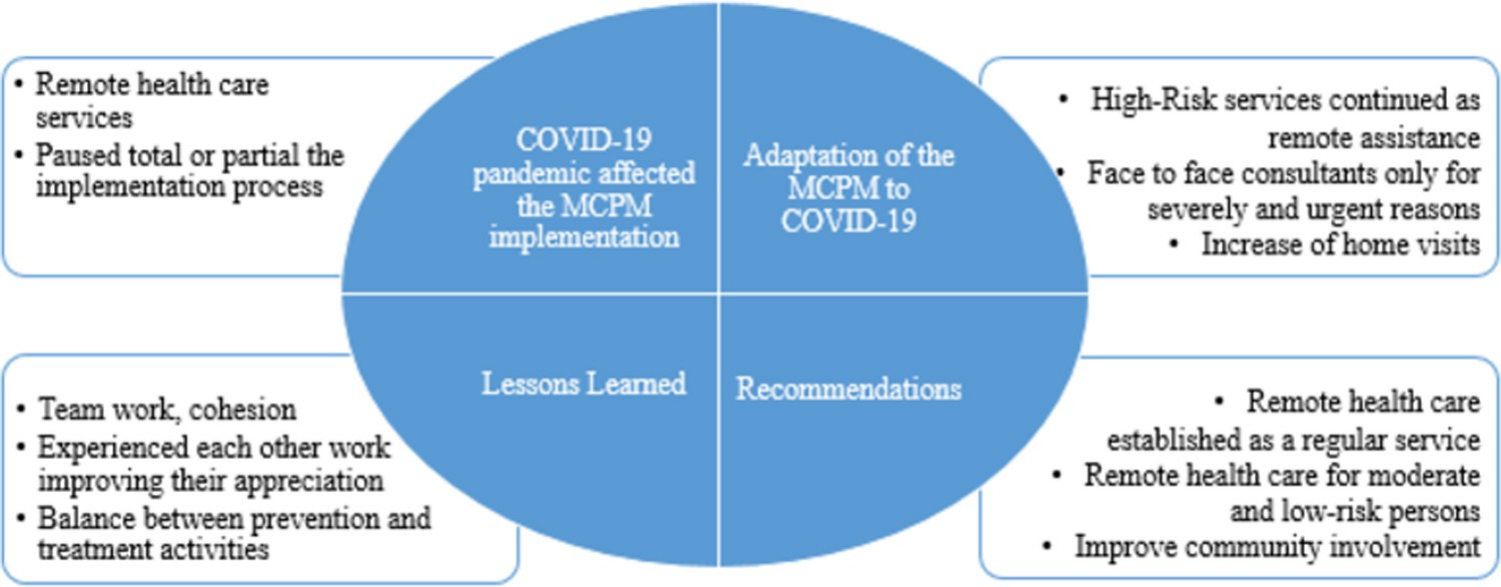
Summary of the main results of the study.

The results are described around four main subjects found in the study: how the COVID-19 pandemic affected the MCPM implementation, how participants adapted the MCPM, lessons learned, and recommendations for its sustainability. They are summarized in Fig 2.

### COVID-19 pandemic affected the MCPM implementation

Several areas of the implementation process were affected positively or negatively by pandemic COVID-19.

The most positive effect was the incorporation of remote health care activities. The empowerment of patients in chronic care took a more appropriate place since they had less contact with the care team. They had to make their own decisions and learn about their chronic illnesses. The directors of the health centers and the case managers supported the continuity of care through telephone assistance. However, contacts inevitably decreased because the nurses in charge had to fulfill other functions related to COVID-19.

*Because despite all this, we have continued to work partially, but we have continued then it tells you about a super good implementation (C3, DM 40)*

*COVID-19 Pandemic crushed us, and we are still at zero. We did not achieve the work we were going to do this year (2020) (C1, DM 79)*

In contrast, the most relevant negative impact was on the abrupt disruption, total or partial, of the MCMP implementation process. According to patient’s risk, intervention with person-centered care focus was dramatically diminished, especially face-to-face care delivery. Consequences include the difficulty of maintaining continuity of care since remote monitoring initially had to be done with personal telephones and without direct access to the clinical record. Communication and consulting with specialists at the secondary level were also hard to maintain.

*I also think that it will not be easy to go back … I feel that the pandemic did us a lot of damage in chronic patients, and it will be a great challenge to rebuild what we had before. It will not be easy. (HT, C3 111)*

*I felt that the pandemic made us change very abruptly because we had built a solid work team, patients had a reference and from one moment to another we had to adapt to the situation, which we were not really prepared for. (HT,C4 81)*

The reorganization of health teams for patients with COVID-19 had substantial implications for providing care for chronic patients, as the number of appointments was drastically reduced, and self-management workshops discontinued. On the other hand, patients began to require care due to decompensation and worsening of their illnesses. This situation stressed the care teams who felt limited to delivering an adequate and timely response.

*It has negatively affected, I would say, many patients are now very decompensated from their NCDs. (HT, C7 82)*

*With the development of the pandemic, the truth is that I feel that we only focused on COVID that is very wrong in terms of the other things we are leaving behind. (DM, C5, 40)*

### Adaptation of the MCPM to COVID-19

The main adaptation that local teams did to the MCPM to face the pandemic was developing and improving remote health care services. Most of the face-to-face services were either suspended or adapted to remote assistance due to total or partial pause in implementing MCPM, keeping care for severely decompensated patients or with urgent problems. For example, in some PHC, high-risk intervention and follow-up were done via phone contacts.

*Changed the delivery and the type of care completely, given remote care via telephone to protect the health of these patients. Obviously, stopped all kinds of in-person care of the MCPM. (HT, C7 70)*

*High-risk intervention I know that there are physicians in charge and connected with the patient making everything by telephone, so the high risk was not abandoned (HT, C4 56)*

Also, they refer that they adapted and incorporated home visits to those who needed urgent face-to-face clinical services, avoiding the risk of going to health services centers.

*We focused only on telephone contact, and home visits were for the most extreme cases. (HT, C5, 62)*

### Lessons learned

Participants mentioned the importance of teamwork, cohesion, and clearly defined roles among their main learnings. The pandemic forced them to constantly reorganize activities, make local adjustments, switch activities, and coordinate the multidisciplinary team to respond to the dynamic demands. It allowed them to experience the roles, activities, and responsibilities, appreciating each other’s work.

*I feel that the pandemic has put us in the role of others. For example, a lot has been restructured here in the center. Many nutritionists, nurses, and managers have had to receive the patients at the centers´ entrance, facing them directly. (HT, C4, 53)*

Another lesson learned is the balance between prevention and treatment activities. Pandemic had required care teams to focus intensely on treating COVID-19 infections. However, they have learned to prioritize the demand to cover extreme and urgent cases and to receive patients with chronic conditions after prolonged lockdowns.

*Seek for a balance between preventives and treatment, which I believe is the goal that we truly have now. (HT, C1, 84)*

### Recommendations

The main recommendation they refer is that remote health services, such as phone consultations, follow up and concealing, should be established as a regular service. COVID-19 pandemic increased the frequency and the coverage of remote clinical services, where the participants refer that they were able to realize the benefits, effectiveness, and diversity offered within the multidisciplinary care team.

*The pandemic facilitated that the rest of the health care team, who had not worked before in remote services, understood its effectiveness. (HT, C6, 61)*

About, MCPM they recommend utilizing phone consultations, follow-ups, and video consultants for moderate and low-risk patients, realizing that it is not always necessary for an in-person follow-up. They emphasize the need for a good internet connection, especially in the more remote areas. In addition, the interviewees mention that the restriction of face-to-face health care made patients begin to inform themselves and learn how to manage their health.

*Tele-attention is already implemented, schedules are accommodated, so I think it should be maintained (HT, C2,60)*

*As for everything that we are going through, the use of telephone, video-calls, distance support of self-management, I believe that it would be relevant to strengthen the technological tools (HT, C2, 76)*

Finally, some participants recommend improving the involvement and activation of the community settings. Schools, community centers, and other areas of the PHC could be used to provide non-COVID-19 infection care services. For example, to persons with multimorbidity and high risk or with severe complications that need to receive face to face care in a low-risk place of COVID-19 infection.

*I recommend that community settings should enable, for example, local headquarters or schools to cover severe patients, to decrease the demand in the PHC, which could help (HT, C6, 113)*

## Discussion

The implemented model has shown positive results in health services utilization [16] and the systematization of this experience t has served as a base for the national scale-up of a similar strategy [13]. This study exhibits the effect of a pandemic on implementing a complex intervention in the health care system. The main findings conclude that PHC abruptly experimented a total or partial pause of the implementation process because of the pandemic. Impacts like this strengthen the resilience of the care team, forcing them to develop strategies that can prioritize and reorganize care in advance in an effective manner [17].

The negative effect of COVI-19 pandemic reflects in the implementation or development of non-COVID-19 related services and activities. However, the positive effect is that care teams experienced work transitions and adjustments, preparing them for another initiative. In any case, this situation is not foreign to the rest of Latin American countries, where their health services have also been interrupted [2, 18].

Remote health care services were described as the main element that the pandemic potentiated and should be continued in the MCPM implementation. Although high-risk intervention strategy had already implemented remote assistance during the early start of the piloting, other health providers were initially resistant to incorporate these services in their daily routine. With the pandemic, the expansion and diversity of remote services were experienced and implemented by the multidisciplinary team, which facilitated its acceptance. The success of telemedicine and remote services has been supported with guidelines and is strongly related to the acceptance of clinical teams, as described in international experiences [19, 20]. Moreover, despite the pandemic evolution, they now support its continuity and sustainability in the MCPM implementation.

In addition, another relevant perception is that with the pandemic, patients were more active in their self-management of chronic diseases. Before the pandemic, workshops and clinical services offered self-management support at the health centers. Nevertheless, now, with the cancelation of face-to-face consultants, patients were encouraged in one way or the other to seek information, learn about, and self-manage their health problems. It meant a breakthrough in a subject that has been difficult to improve for health teams in recent years. Despite self-care during the pandemic management of stress is described in the literature [21], today, patients have had to become active and begin to take more significant participation in the care of their health as a pandemic consequence [22, 23].

The study’s strengths are that the data collection process was comprehensive and included vital profiles, and data reached high saturation levels. Although the interviews were done six months after the start of the pandemic in Chile, the study implemented innovative remote strategies to collect data and reach the process goal. In addition, a solid theoretical backup [14] allowed the study’s design and organized the provided information appropriately.

Some of the limitations found were the high rotation of health practitioners, including some persons that have not been in the implementation process of the MCPM. Therefore, the recruitment of participants was difficult, but we could reach enough participants for consistent data saturation. Furthermore, the qualitative studies’ proper bias was also a limitation and a strength generating an extended version from the participants. The involvement of decision-makers of the SSMSO could have influenced as an authority on the interviewer’s answers, though performed separate group interviews.

Further studies would be appropriate to complement the results found in this study. Perception from the patients would add information to complete the perspective from both internal and external users. Also, a comparison of the perspective with the impact in health services utilization would give enough support for the continuity of the MCPM in the health system. Finally, when the pandemic decreases its intensity and allows the reactivation of other non-COVID-19 health services, it would be interesting to describe the process of reactivation of the MCPM.

## Conclusion

The pandemic has forced the dismantling of the traditional care system and forced it to place once adjusted to the new demands. This focus on one big problem is proving unsustainable over time. However, it has been necessary and crucial to reorganize the care system to resume its normal functions under different conditions. It is an opportunity to install and strengthen person-centered care integrating innovations and technologies that have been already in frequent use during the pandemic. In this context, strategies such as remote health care can be consolidated and contribute to the recovery of comprehensiveness and continuity of care, combining face-to-face and remote activities according to people’s risk.
